# Proton Pump Inhibitor Use Following Esophageal Variceal Ligation and Its Impact on Clinical Outcomes: Real-World Data from the TriNetX Global Collaborative Network

**DOI:** 10.3390/diagnostics15131653

**Published:** 2025-06-28

**Authors:** Nisar Amin, Harleen Chela, Muhammad Faisal Mubarak, Mark Ayoub, Ebubekir Daglilar

**Affiliations:** 1 Department of Internal Medicine, Charleston Area Medical Center, Charleston, WV 25304, USA; 2 Division of Gastroenterology and Hepatology, Charleston Area Medical Center, Charleston, WV 25304, USA; harleen.chela@hsc.wu.edu (H.C.); ebubekir.daglilar@hsc.wvu.edu (E.D.); 3 Division of Gastroenterology, Department of Medicine, College of Medicine, King Saudi University, Riyadh 11451, Saudi Arabia

**Keywords:** cirrhosis, gastrointestinal bleeding, esophageal varices, proton pump inhibitors, variceal band ligation

## Abstract

**Background:** Proton pump inhibitors (PPIs) are frequently used after endoscopic variceal ligation (EVL) to reduce post-procedural bleeding, though studies have shown mixed results regarding their efficacy. While some suggest benefits, others report no significant advantage and highlight potential risks, including infection, kidney injury, and hepatic complications in cirrhotic patients. This study utilizes the TriNetX global health research network to evaluate the outcomes of PPI use following elective EVL for primary prophylaxis. **Methods:** This retrospective cohort study was conducted using the TriNetX database to evaluate adult patients with cirrhosis and esophageal varices who underwent EVL for primary prophylaxis. Patients who received at least two weeks of PPI therapy following EVL were compared to those who did not receive PPI within one month post-procedure. Outcomes assessed included esophageal bleeding, adverse events such as acute kidney injury (AKI), pneumonia, spontaneous bacterial peritonitis (SBP), *Clostridioides difficile* infection, hepatic encephalopathy, and all-cause mortality at 4 weeks and 8 weeks. **Results:** Of 6196 patients with cirrhosis and esophageal varices who underwent EVL, 12% (*n* = 764) received adjuvant PPI post-procedure, while 88% (*n* = 5432) did not receive PPI. After 1:1 propensity score matching, two well-balanced cohorts of 618 patients each were analyzed. PPI use was not associated with a reduction in esophageal bleeding at either 4 weeks (1.8% vs. 1.7%, *p* = 0.89) or 8 weeks (2.3% vs. 1.9%, *p* = 0.60). However, the composite adverse event rate—including SBP, hepatic encephalopathy, pneumonia, *C. difficile*, and acute kidney injury (AKI)—was significantly higher in the PPI group at both 4 weeks (7.9% vs. 3.0%, *p* < 0.01) and 8 weeks (13.2% vs. 3.0%, *p* < 0.01). Subgroup analysis showed no significant differences in pneumonia, SBP, or *C. difficile* infection at either time point. Hepatic encephalopathy was significantly more frequent in the PPI group at 8 weeks (4.9% vs. 2.0%, *p* = 0.01), and AKI occurred more often at both 4 weeks (5.7% vs. 2.0%, *p* < 0.01) and 8 weeks (9.6% vs. 2.1%, *p* < 0.01). Mortality was similar at 4 weeks but significantly higher in the PPI group at 8 weeks (4.3% vs. 1.7%, *p* < 0.01). **Conclusions:** PPI use after prophylactic EVL did not reduce bleeding risk and was linked to higher rates of adverse events. These findings suggest routine use may not be beneficial and should be reconsidered in cirrhotic patients who undergo EVL for primary prophylaxis.

## 1. Introduction

Esophageal varices (EVs) arise as a consequence of elevated portal venous pressure, most commonly due to portal hypertension secondary to cirrhosis, particularly in Western populations. The prevalence of varices increases with the severity of liver disease, with up to 85% of patients with decompensated cirrhosis exhibiting varices [1].

Variceal hemorrhage represents one of the most life-threatening complications of cirrhosis, with a reported two-year incidence of 20–30% and associated mortality rates of 6–8% [2,3]. The risk of bleeding is proportional to the size of the varices and is further augmented by the presence of red wale signs, bacterial infections, and a hepatic venous pressure gradient (HVPG) exceeding 10 mmHg [4,5]. Following an initial bleed, the likelihood of rebleeding surpasses 60% within one year [6].

Current guidelines recommend that patients with cirrhosis—particularly those with a liver stiffness measurement (LSM) ≥ 20 kPa and a platelet count < 150,000/mm^3^—undergo routine surveillance endoscopy to assess for the presence of esophageal varices [7,8]. Management strategies are guided by the stage of cirrhosis and the severity of portal hypertension and encompass both pharmacological and procedural interventions aimed at reducing portal pressure and preventing variceal hemorrhage. These include nonselective beta-blockers (NSBBs), endoscopic variceal ligation (EVL), endoscopic injection sclerotherapy (EIS), and transjugular intrahepatic portosystemic shunt (TIPS) [8].

NSBBs are considered first-line therapy for the prevention and management of variceal bleeding by reducing splanchnic blood flow and, in the case of carvedilol, promoting intrahepatic vasodilation [9,10]. Nevertheless, up to 30% of patients with high-risk varices may have contraindications to NSBB therapy. For patients who are not candidates for or cannot tolerate NSBBs, EVL serves as a second-line modality for both primary and secondary prophylaxis [11,12,13]. While EVL effectively eradicates varices, it does not address the underlying pathophysiological mechanisms of portal hypertension and is associated with complications such as post-ligation hemorrhage, esophageal stricture, dysphagia, chest pain, and odynophagia. Among these, post-ligation ulceration and bleeding are the most clinically significant, with incidence rates reaching up to 15%, and in some cases, resulting in fatal outcomes [14,15,16,17].

Proton pump inhibitors (PPIs) have been used as adjuvant therapy following EVL to minimize the risk of post-EVL bleeding. However, studies have yielded conflicting results regarding their efficacy. Some studies have demonstrated benefits, including reducing the occurrence of early bleeding and extending the rebleeding interval [18,19,20,21]. Conversely, other studies have shown no significant effect of PPI use on the prophylaxis or treatment of esophageal varices or in reducing bleeding post-EVL [22,23,24,25].

Meanwhile, PPI use is associated with adverse effects, including an increased risk of infections such as pneumonia and *Clostridium difficile* infection, as well as acute kidney injury. In cirrhotic patients, PPIs are also linked to an increased risk of hepatic encephalopathy, spontaneous bacterial peritonitis (SBP), and mortality [26,27,28,29,30]. Due to these risks, the Baveno VII guidelines recommend discontinuing PPI therapy in patients who were already on it at the time of EVL; however, they do not provide specific recommendations regarding de novo PPI initiation post-EVL [7].

Overall, the literature on the role of PPIs as adjuvant therapy for post-EVL bleeding prevention is limited to small cohort studies with contradictory findings. In this retrospective study, we used the global TriNetX health research network database to evaluate the outcomes of PPI use, including esophageal bleeding, AKI, pneumonia, SBP, *Clostridioides difficile* infection, hepatic encephalopathy, and all-cause mortality in cirrhotic patients following elective EVL for primary prophylaxis.

## 2. Materials and Methods

This retrospective cohort study leveraged data from the Global Collaborative Network, a TriNetX Research Network that integrates longitudinal medical records from 120 healthcare organizations across 30 countries [31]. With access to de-identified records from over 270 million patients, the database provides a comprehensive collection of patient demographics, clinical diagnoses, procedures, prescribed medications, laboratory results, and healthcare utilization patterns. This extensive dataset ensures compliance with data privacy regulations while facilitating robust and large-scale analytical research.

The database was queried to identify adults (≥18 years) diagnosed with cirrhosis and esophageal varices. Patients were included if they had a first-time diagnosis of esophageal varices and underwent outpatient elective EVL for primary prophylaxis.

Patients with a diagnosis of peptic ulcer disease, a history of esophageal variceal bleeding, and those receiving nonselective beta-blocker therapy were excluded from the study. Additionally, patients with a bilirubin level greater than 4 mg/dL were excluded, as matching bilirubin levels between the two groups during propensity score matching was not feasible for patients with higher bilirubin levels.

The ICD-10 and CPT codes used for patient identification, along with the detailed inclusion and exclusion criteria, are provided in Supplementary Materials.

Two cohorts were created. Cohort one (treatment) included patients who met the inclusion and exclusion criteria and received at least two weeks of PPI therapy following EVL. Cohort two (control) included patients who met the inclusion and exclusion criteria but did not receive any PPI within 4 weeks of the procedure.

Propensity score matching (1:1 nearest neighbor) was performed to balance baseline characteristics and comorbidities between the two cohorts. Matching variables included age, race, ethnicity, hypertension, heart failure, chronic ischemic heart disease, diabetes mellitus, chronic obstructive pulmonary disease (COPD), and chronic kidney disease (CKD). Individual components of the Model for End-Stage Liver Disease (MELD) score and Child–Turcotte–Pugh (CTP) classification—such as creatinine, bilirubin, INR, sodium, albumin, and presence of ascites—were also included. Cirrhosis etiologies, including alcohol use, nonalcoholic steatohepatitis (NASH), and nonalcoholic fatty liver disease (NAFLD), were considered as well. Additional variables included gastroesophageal reflux disease (GERD), gastritis, portal vein thrombosis, neoplasms, tobacco use, alcohol abuse, and the use of anticoagulant and antithrombotic agents.

Statistical significance was set at *p* < 0.05 with 95% confidence intervals. Kaplan–Meier analysis was employed to assess outcomes and survival probability.

**Figure 1:**
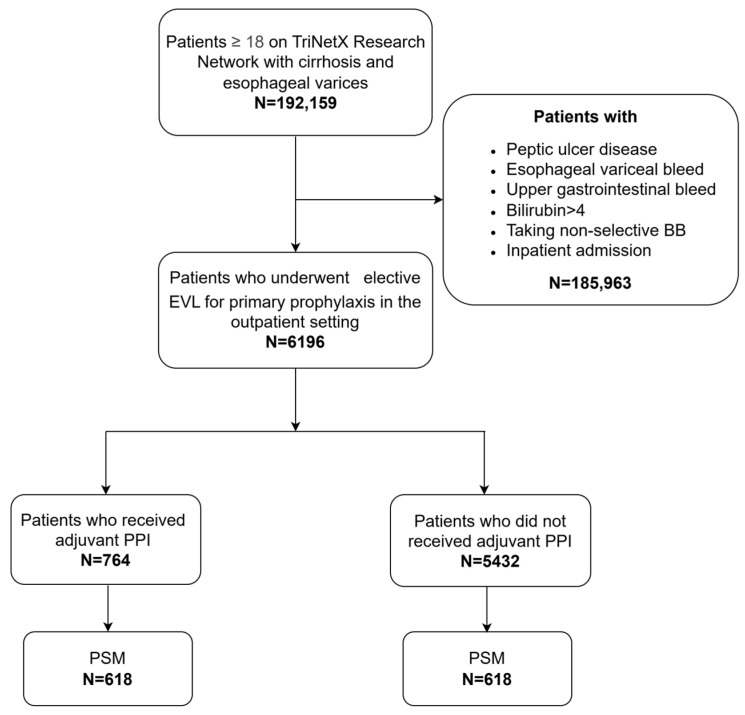
BB: beta blocker. EVL: endoscopic variceal ligation. PPI: proton pump inhibitor. PSM: propensity score matching.

The primary outcome was esophageal variceal bleeding within four weeks and eight weeks post-EVL. The secondary outcomes included PPI-associated adverse effects such as pneumonia, *Clostridium difficile* infection (*C. diff*), spontaneous bacterial peritonitis (SBP), hepatic encephalopathy, and acute kidney injury, as well as mortality within four weeks and eight weeks post-EVL. The study flow diagram is presented in Figure 1.

## 3. Results

A total of 6196 patients with a diagnosis of cirrhosis and esophageal varices who underwent outpatient endoscopic variceal ligation (EVL) were included in this analysis. Of these, 12% (*n* = 764) received adjuvant proton pump inhibitor (PPI) therapy for at least two weeks following EVL, while 88% (*n* = 5432) did not receive any PPI treatment within four weeks of EVL. Two well-matched cohorts (618 patients each) were generated using 1:1 propensity score matching (PSM).

Before matching, patients in the PPI group were, on average, older and had higher rates of several comorbidities compared to the non-PPI group, including hypertensive disease, heart failure, chronic ischemic heart disease, chronic kidney disease, chronic obstructive pulmonary disease, diabetes mellitus, neoplasms, portal vein thrombosis, gastritis, and gastroesophageal reflux disease. Additionally, more patients in the PPI group were receiving hemodialysis. They also had higher rates of ascites, chronic viral hepatitis, nonalcoholic steatohepatitis, nonalcoholic fatty liver disease, alcohol use disorder, and tobacco use. Furthermore, patients in the PPI group had higher use of anticoagulants and antiplatelet agents, along with higher baseline laboratory values for creatinine.

Conversely, patients in the non-PPI group had a higher body mass index, baseline serum sodium, and serum albumin levels. Both groups showed similar baseline bilirubin levels.

Propensity score matching was performed based on baseline characteristics, including demographics, comorbidities, alcohol and tobacco use, ascites, and the etiology of liver cirrhosis, including infection, alcohol-related liver disease, nonalcoholic steatohepatitis (NASH), and nonalcoholic fatty liver disease (NAFLD), and individual components of the MELD and Child–Pugh scores. Post-matching, no significant differences in baseline characteristics were observed between the PPI and non-PPI cohorts. A detailed comparison of baseline variables before and after matching is provided in Table 1.

### 3.1. Primary Outcomes

The primary outcomes of the study were the incidence of esophageal variceal bleeding within 4 weeks and 8 weeks following EVL. Two well-matched cohorts of 618 patients each were established using a 1:1 propensity score matching (PSM) model. Post-matching analysis demonstrated that patients who received adjuvant PPI therapy for two weeks post EVL showed no significant difference in the rates of esophageal bleeding at both 4 weeks (1.8% vs. 1.7%, *p* = 0.89) and 8 weeks (2.3% vs. 1.9%, *p* = 0.60) compared to those who did not receive PPIs.

These findings suggest that PPI therapy does not confer a benefit in promoting esophageal ulcer healing following EVL.

**Figure 2:**
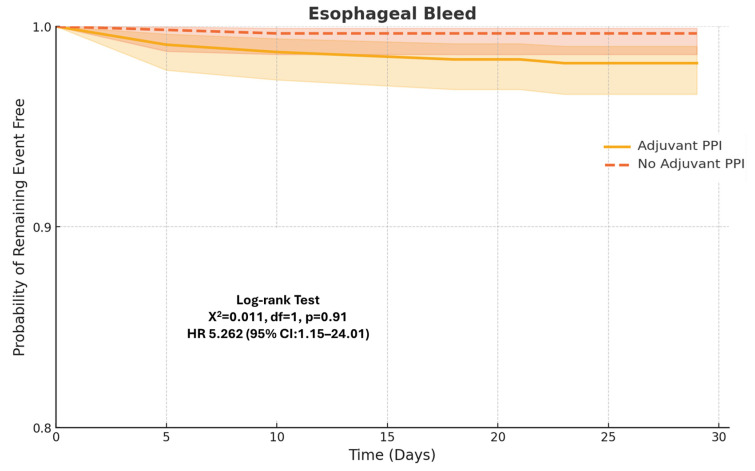
Kaplan–Meier estimates of esophageal bleeding within 4 weeks of variceal ligation.

Kaplan–Meier survival curves depicting bleeding outcomes over time are shown in Figure 2.

### 3.2. Secondary Outcomes

The secondary outcomes of the study included adverse events—spontaneous bacterial peritonitis (SBP), hepatic encephalopathy, pneumonia, *Clostridioides difficile* infection (*C. difficile*), and acute kidney injury (AKI)—as well as all-cause mortality. The composite outcome of SBP, hepatic encephalopathy, pneumonia, *C. difficile*, and AKI was significantly higher in the PPI-treated group at both 4 weeks (7.9% vs. 3.0%, *p* < 0.01) and 8 weeks (13.2% vs. 3.0%, *p* < 0.01).

Subgroup analysis revealed no significant differences in the rates of SBP between the two groups at 4 weeks (1.9% vs. 1.7%, *p* = 0.79) or 8 weeks (3.2% vs. 1.8%, *p* = 0.16). *C. Difficile* infection rates were also similar at 4 weeks (1.7% vs. 1.7%, *p* = 0.97) and 8 weeks (1.8% vs. 1.8%, *p* = 0.96). Pneumonia rates were higher in the PPI-treated group, but this difference was not statistically significant at either 4 weeks (2.3% vs. 1.8%, *p* = 0.61) or 8 weeks (3.4% vs. 1.9%, *p* = 0.14).

Hepatic encephalopathy occurred at similar rates at 4 weeks (3.3% vs. 1.8%, *p* = 0.10) but was significantly higher in the PPI group at 8 weeks (4.9% vs. 2.0%, *p* = 0.01). AKI was significantly more common in the PPI group at both 4 weeks (5.7% vs. 2.0%, *p* < 0.01) and 8 weeks (9.6% vs. 2.1%, *p* < 0.01).

**Figure 3:**
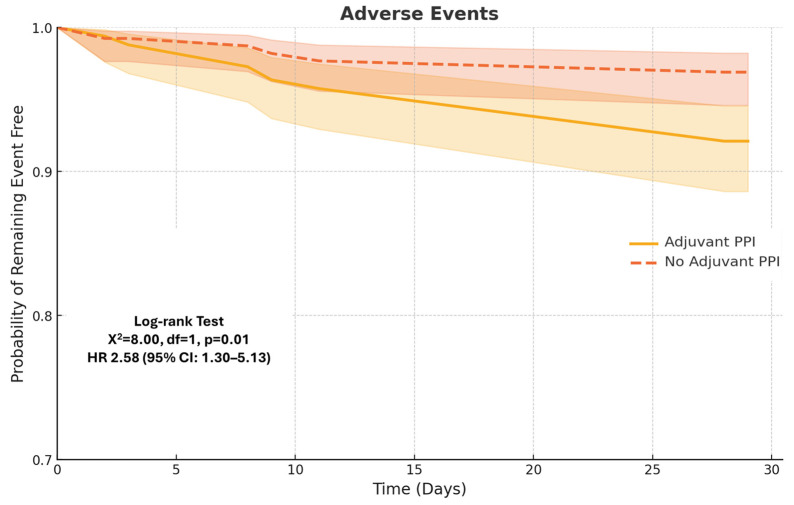
Kaplan–Meier estimates of adverse events, a composite of AKI, hepatic encephalopathy, SBP, pneumonia, *C. difficile*, and AKI.

Composite outcomes are illustrated in Figure 3.

**Figure 4:**
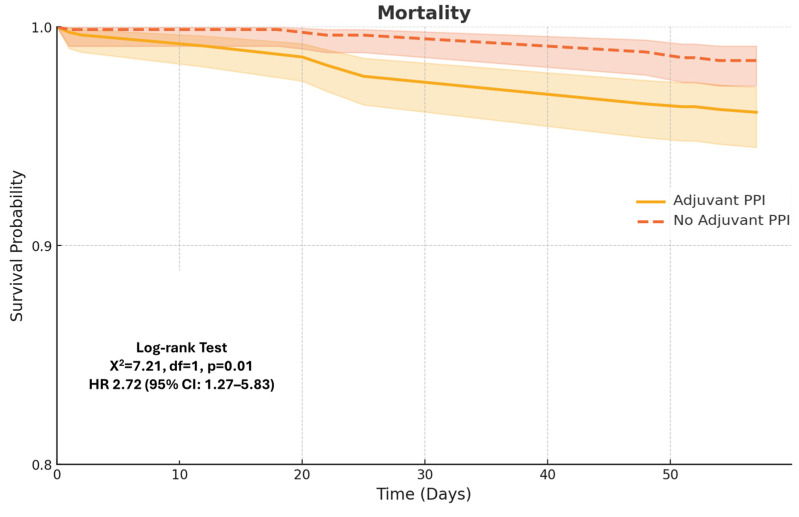
Kaplan–Meier estimates of mortality within 8 weeks of esophageal variceal ligation.

The mortality rate was comparable between the two groups at 4 weeks (2.4% vs. 1.6%, *p* = 0.31); however, at 8 weeks, it was significantly higher in the PPI-treated group (4.3% vs. 1.7%, *p* < 0.01). Outcomes are presented in Figure 4.

## 4. Discussion

Elective endoscopic variceal ligation (EVL) is a standard intervention for the treatment and prophylaxis of esophageal variceal bleeding in patients with cirrhosis. While EVL is effective in obliterating varices through mechanical strangulation, it can lead to post-ligation mucosal ulceration, which carries a risk of delayed bleeding [12]. Proton pump inhibitors (PPIs) have been proposed as adjunctive therapy to enhance ulcer healing and reduce complications following EVL. Although PPIs are well-established in promoting the healing of gastric ulcers and gastric varices via acid suppression, their efficacy in facilitating esophageal mucosal healing—particularly after EVL-induced injury—remains unclear. Meanwhile, growing concerns have emerged regarding the potential adverse effects of PPI use in both the general population and in patients with cirrhosis.

In this large global cohort study, we found that the use of at least two weeks of PPI therapy following EVL for primary prophylaxis—among patients without a prior history of esophageal bleeding, gastrointestinal bleeding, or peptic ulcer disease—was not associated with a significant reduction in post-EVL bleeding rates compared to patients who did not receive PPIs. This finding aligns with results from smaller cohort studies. For example, Shaheen et al. compared two cohorts of 22 patients—one treated with pantoprazole for nine days post-EVL and the other without PPI therapy—and found smaller ulcer sizes in the PPI group but no significant differences in ulcer number or post-ligation bleeding rates [22]. Similarly, Jang et al. observed no significant difference in bleeding rates between PPI and non-PPI groups following endoscopic variceal obturation (EVO), although the PPI-treated cohort demonstrated a longer rebleeding interval [21]. In contrast, Hidaka et al. conducted a randomized trial in Japan involving 43 patients undergoing either primary or secondary prophylactic EVL and reported a significantly higher risk of post-EVL bleeding in the non-PPI group, despite comparable variceal recurrence rates [19]. Likewise, Khawaja et al. identified an increased bleeding risk in patients not treated with PPIs after EVL [20]. It is important to note that these latter studies included patients with active or prior bleeding and those receiving secondary prophylaxis, whereas our study focused exclusively on patients with esophageal varices undergoing primary prophylaxis. Additionally, regional differences in patient populations across studies may have contributed to the variability in outcomes.

Our study also found that PPI use following EVL was associated with a significantly increased risk of acute kidney injury (AKI), with the incidence more than fourfold higher compared to the non-PPI group. This finding is consistent with prior studies linking PPIs to AKI. While the exact mechanism remains unclear, proposed pathways include oxidative stress, immune and inflammatory responses, mitochondrial dysfunction, calcium overload, and tubular cell necrosis [32]. Several studies have identified a strong correlation between PPI use and acute interstitial nephritis, a mechanism contributing to PPI-associated AKI. A case-control study by Blank et al. reported an increased rate of acute interstitial nephritis among current PPI users [33]. Similarly, a review by Sierra et al. identified a low but notable prevalence of PPI-associated interstitial nephritis [34]. The association between short-term PPI use and AKI in our cohort may be explained by this adverse renal effect, which often manifests within days to weeks of exposure.

In addition, PPI use in our study was associated with a significantly higher risk of hepatic encephalopathy (HE), particularly at the 8-week follow-up. This is consistent with prior research. Dam et al. observed a 72% increased risk of spontaneous bacterial peritonitis (SBP) and an 88% increased risk of HE among cirrhotic patients on PPIs [35]. Nardelli et al. similarly demonstrated that PPI use was associated with both minimal and overt HE [26], and a population-based study from Taiwan found a dose-dependent relationship between PPI exposure and HE risk [27]. Several mechanisms have been proposed for the development of SBP and HE associated with PPI use, including alterations in intestinal flora, bacterial translocation, small bowel bacterial overgrowth, reduced microbial diversity, and increased prevalence of Streptococcaceae [36].

PPIs have also been associated with an increased risk of infections, including SBP, *C. difficile* infection (CDI), and pneumonia. Mahmud et al. reported a 77% higher hazard of developing SBP among PPI users [37]. A population-based study by Maret-Ouda et al. from Sweden demonstrated a significantly increased risk of pneumonia in PPI users [38]. A meta-analysis of 56 studies by Trifan et al. found that PPI use was associated with nearly double the odds of developing CDI [39]. Potential mechanisms for this association include the elevation of gastric pH, which allows vegetative forms of *C. difficile* to survive [40], and alterations to gut microbiota, favoring colonization by pathogenic organisms such as Enterococcus and Streptococcus [41]. In our study, although rates of *C. difficile*, pneumonia, and SBP were higher in the PPI group, these differences did not reach statistical significance. This may be attributable to the relatively short follow-up period, as longer exposure may be necessary for microbiota alteration and subsequent infection risk. Further investigation with extended follow-up is warranted.

Regarding overall mortality, we found no significant difference between groups at the 4-week follow-up. However, by 8 weeks, mortality was significantly higher among PPI users. This finding is consistent with prior studies. Nardelli et al. reported a survival rate of 41% in cirrhotic patients using PPIs versus 81% in nonusers over a defined follow-up period [26]. Kwon et al. similarly found that PPI use was independently associated with increased mortality, regardless of liver disease severity [42]. The increased mortality observed in our study may reflect the cumulative burden of PPI-associated complications, including AKI and HE—both significantly more frequent in the PPI group—as well as increased but nonsignificant rates of SBP, CDI, and pneumonia.

Finally, data from noncirrhotic populations undergoing esophageal mucosal interventions also cast doubt on the benefits of PPI therapy. Several large cohort studies have shown that PPIs do not significantly improve ulcer healing, reduce bleeding, or enhance outcomes following esophageal endoscopic submucosal dissection (ESD) for superficial esophageal squamous cell carcinoma [43,44]. These findings further question the utility of routine PPI use following esophageal mucosal injury, including after EVL.

### Strength and Limitation

To the best of our knowledge, this is the largest study to date evaluating the role of proton pump inhibitors (PPIs) as adjuvant therapy following EVL. The inclusion of a large, multinational cohort enhances the external validity of our findings and supports their applicability across diverse patient populations. The substantial sample size strengthens the precision of our outcome estimates and enables robust subgroup analyses.

To minimize potential bias, we employed propensity score matching (PSM) to generate well-balanced comparison groups and reduce the impact of confounding variables. Nevertheless, several limitations must be acknowledged. As with all retrospective studies, the observational design carries an inherent risk of residual confounding. Additionally, the lack of granular data regarding the specific types, dosages, and durations of PPI therapy restricts our ability to assess potential dose-response effects or compare outcomes across individual PPI agents. Moreover, the exclusion of patients with bilirubin levels >4 mg/dL limits the generalizability of our findings to this subgroup of patients.

In addition, while the database does not allow calculation of Model for End-Stage Liver Disease (MELD) or Child–Pugh Class (CPC) scores directly, we incorporated the individual laboratory parameters that constitute these scoring systems into the PSM model. Consequently, we believe the matched cohorts were adequately balanced in terms of liver disease severity.

## 5. Conclusions

In this large global cohort study, we evaluated the impact of PPI use as adjuvant therapy in patients undergoing prophylactic EVL. Our findings indicate that while PPI use did not reduce the risk of post-EVL bleeding, it was associated with a significantly higher incidence of adverse events, including acute kidney injury, infections, and hepatic decompensation. These results suggest that routine PPI use following EVL in cirrhotic patients with esophageal varices may not be beneficial and should be approached with caution.

## Figures and Tables

**Table 1:** Baseline Characteristics of Study Cohorts Before and After Propensity Matching.

	Before Propensity Score Matching ^a^			After Propensity Score Matching ^a^		
	PPI (*n* = 766)	No-PPI (*n* = 6196)	*p*-Value	PPI (*n* = 618)	No-PPI (*n* = 618)	*p*-Value
Demographics						
Age at Index, Mean years ± SD	60.6 ± 11.7	59.6 ± 12.0	0.04	60.6 ± 11.7	60.3 ± 11.7	0.66
Female	238 (38.1)	2068 (39.6)	0.01	237 (38.3)	245 (39.6)	0.64
Male	383 (61.3)	3028 (58.0)	0.12	377 (61.0)	370 (59.9)	0.68
Race						
White	420 (67.2)	3946 (75.6)	0.01	418 (67.6)	434 (70.2)	0.33
Black or African American	39 (6.2)	210 (4.0)	0.01	38 (6.1)	41 (6.6)	0.73
Ethnicity						
Not Hispanic or Latino	453 (72.5)	3341 (64.0)	0.01	366 (59.2)	375 (60.7)	0.60
Hispanic or Latino	92 (14.7)	916 (17.6)	0.08	91 (14.7)	81 (13.1)	0.41
Comorbidities						
Hypertensive disease	371 (59.4)	2262 (43.3)	0.01	366 (59.2)	375 (60.7)	0.60
Heart failure	72 (11.5)	356 (6.8)	0.01	68 (11.0)	74 (12.0)	0.59
Chronic ischemic heart disease	148 (23.7)	704 (13.5)	0.01	145 (23.5)	135 (21.8)	0.50
COPD	71 (11.4)	343 (6.6)	0.01	71 (11.5)	78 (12.6)	0.54
CKD	128 (20.5)	451 (8.6)	0.01	126 (20.4)	115 (18.6)	0.43
Diabetes mellitus	283 (45.3)	1708 (32.7)	0.01	279 (45.1)	277(44.8)	0.90
Neoplasms	327 (52.3)	1831 (35.1)	0.01	321 (51.9)	316 (51.1)	0.78
Portal vein thrombosis	76(12.2)	361 (6.9)	0.01	75 (12.1)	79 (12.8)	0.730
Gastritis	66 (10.6)	273 (5.2)	0.01	62 (10.0)	59 (9.5)	0.77
Gastroesophageal reflux disease	281(45.0)	1236 (23.7)	0.01	274 (44.3)	283 (45.8)	0.61
Ascites	351 (56.2)	1994 (38.2)	0.01	345 (55.8)	343 (55.5)	0.91
Tobacco use	51 (8.2)	213 (4.1)	0.01	51 (8.3)	60 (9.7)	0.37
Alcohol abuse	105 (16.8)	601 (11.5)	0.01	102 (16.5)	107 (17.3)	0.70
Chronic viral hepatitis	151 (24.2)	1009 (19.3)	0.01	148 (23.9)	133 (21.5)	0.0.31
NASH	143 (22.9)	812 (15.6)	0.01	140 (22.7)	145 (23.5)	0.74
NAFLD	173 (27.7)	1092 (20.9)	0.01	170 (27.5)	186 (30.1)	0.32
BMI	29.1 ± 6.8	29.9 ± 6.8	0.01	29.0 ± 6.7	29.9 ± 7.4	0.06
Hemodialysis	13 (2.1)	28 (0.5)	0.01	12 (1.9)	11 (1.9)	0.83
Medication						
Long-term (current) use of anticoagulants and antithrombotic/antiplatelets	84 (13.4)	311 (6.0)	0.01	81 (13.1)	87 (14.1)	0.62
Lab Results						
Bilirubin	1.7 ± 1.7	1.6 ± 1.7	0.14	1.7 ± 1.7	1.6 ± 1.4	0.38
Sodium	137.1 ± 4.1	137.9 ± 3.6	0.01	137.1 ± 4.1	137.3 ± 3.7	0.50
Creatinine	1.1 ± 1.0	0.9 ± 0.7	0.01	1.1 ± 1.0	1.1 ± 1.0	0.32
Albumin	3.4 ± 0.6	3.5 ± 0.6	0.01	3.4 ± 0.6	3.4 ± 0.6	0.12
INR	1.31 ± 0.33	1.26 ± 0.30	0.01	1.31 ± 0.33	1.29 ± 0.34	0.37

^a^ Values are the mean ± SD or *n* (%) unless otherwise specified. BMI, body mass index; CAD, coronary artery disease; CKD, chronic kidney disease; COPD, chronic obstructive pulmonary disease; INR, international normalized ratio; NASH, nonalcoholic steatohepatitis; NAFLD, nonalcoholic fatty liver disease. In the demographics section, the ‘Other’ category for gender, race, and ethnicity included a minimal number of patients with no significant intergroup differences and was therefore not reported.

## Data Availability

The data analyzed in this study are included in the published article. Access to additional data is restricted and may be granted upon request, subject to third-party data sharing policies.

## Supplementary Materials

The following supporting information can be downloaded at: https://www.mdpi.com/article/10.3390/diagnostics15131653/s1, Table S1. Inclusion criteria. Table S2. Exclusion criteria. Table S3. Propensity score matching inputs.
