# Supplementary material for: Proton Pump Inhibitor Use Following Esophageal Variceal Ligation and Its Impact on Clinical Outcomes: Real-World Data from the TriNetX Global Collaborative Network
Source: Diagnostics (Basel). 2025 Jun 28;15(13):1653. doi: 10.3390/diagnostics15131653 (PMC12248837; doi:10.3390/diagnostics15131653)
Supplement: Supplementary file 1 [file diagnostics-15-01653-s001.zip › diagnostics-3661575-supplementary.pdf]

SUPPLEMENTAL

Table S1. Inclusion criteria

**Inclusion criteria**	**ICD -10, CPT, SNOMED codes**
Age>18, Male, Female	
Fibrosis and cirrhosis of liver	K74
Esophageal varices	I85
Esophagogastroduodenoscopy with ligation	43244, 43400, 43205, 67364009
Proton pump inhibitor (prescribed 14 days or more after the endoscopic variceal ligation, in the treatment group)	114979, 17128, 816346, 7646, 283742, 40790

Table S2. Exclusion criteria

**Exclusion criteria**	**ICD -10, CPT, SNOMED, RxNorm codes**
Gastrointestinal hemorrhage (any instance before endoscopic variceal ligation)	K92.2
Melena (any instance before endoscopic variceal ligation)	K92.1
Hematemesis (any instance before endoscopic variceal ligation)	K92.0
Peptic ulcer disease (any instance before endoscopic variceal ligation)	K27
Nonspecific beta blockers (any instance within 6 months on or before endoscopic variceal ligation)	8787, 20352, 7226
Inpatient encounter	-
Bilirubin>4 mg/dL	9050

Table S3. Propensity score matching inputs

Covariates	ICD -10, CPT, SNOMED codes
Demographics	M, F, 2186-5, 2106-3, 2135-2, 2054-5, 2131-1, 2028-9
Hypertensive diseases	I10-11A
Heart failure	I50
Chronic obstructive disease	J44
Chronic kidney disease	N18.3, N18.4, N18.5,
Diabetes mellitus	E08-E13
Ascites	R18
Chronic ischemic heart disease	I25
BMI	9083
Gastroesophageal reflex disease	K21
Gastritis without bleeding	K29.70
Tobacco use	Z72.0
Alcohol abuse	F10.10
Portal vein thrombosis	I81
Neoplasm	C00-D49
Hemodialysis	1012752
Anticoagulation use	Z79.0
Chronic viral hepatitis	B18
Nonalcoholic steatohepatitis	K75.81
Alcoholic liver disease	
Bilirubin in serum	9050
Sodium in serum	9029
Creatinine in serum	9024
Albumin in serum	9045
INR in plasma	9032
